# Occurrence of Heavy Metals in Sediments and Bioaccumulation Factor in *Rangia cuneata* Clams from a Protected Coastal Lagoon in Mexico

**DOI:** 10.3390/toxics14050411

**Published:** 2026-05-09

**Authors:** Alejandro Ruiz-Marin, Claudia Alejandra Aguilar-Ucan, Carlos Montalvo-Romero, Julia G. Cerón-Breton, Francisco Anguebes-Franseschi

**Affiliations:** Department of Environmental Engineering, Faculty of Chemistry, Autonomous University of Carmen, Calle 56, No. 4, Av. Concordia, Col. Benito Juárez, Ciudad del Carmen, Campeche C.P. 24180, Mexico; caguilar@pampano.unacar.mx (C.A.A.-U.); cmontalvo@pampano.unacar.mx (C.M.-R.); jceron@pampano.unacar.mx (J.G.C.-B.); fanguebes@pampano.unacar.mx (F.A.-F.)

**Keywords:** Terminos Lagoon, *Rangia cuneata*, heavy metals, bioaccumulation, ecological risk, seasonal variation

## Abstract

This study evaluated the seasonal variability, origin, and ecological risk of heavy metals in the Pom–Atasta lagoon system, a tropical estuary in southeastern Mexico subject to increasing anthropogenic pressure. The main objective was to determine how seasonal changes influence the distribution, bioavailability, and risk of metals in sediments and benthic organisms. Thirty sampling stations were monitored during dry, rainy, and north wind seasons. Sediment concentrations of As, Cd, Cr, Ni, Pb, and V were measured, and bioaccumulation was assessed in the bivalve *Rangia cuneata*. Ecotoxicological risk was evaluated using the Adverse Effects Index (AEI), Toxic Risk Index (TRI), and potential ecological risk index (ERI). The results showed higher metal concentrations during the rainy and north wind seasons, likely due to increased runoff and sediment resuspension. Cr and Ni exhibited the highest enrichment, with values from 115.0 to 130.4 µg g^−1^ and from 60.5 to 75.9 µg g^−1^, respectively. Ni showed the highest bioaccumulation factor (BSAF > 1.51) in *R. cuneata*, indicating high mobility and environmental availability. Weak correlations among some metals (As, Cr, and Pb) suggest mixed natural and anthropogenic sources. TRI values indicated low to moderate toxic risk, and ERI classified most sites as low risk (ERI < 60) at several stations. Organic carbon levels remained within tolerable limits (<10%) for benthic fauna. These findings highlight the role of seasonal dynamics in metal distribution and confirm *R. cuneata* as a suitable bioindicator for monitoring ecological health in tropical estuarine systems.

## 1. Introduction

Many trace metals have been recognized as essential for metabolic processes in microorganisms; however, some of these metals can become toxic to biota above a specific threshold concentration and can become a threat to the environment. The enrichment of metals in coastal ecosystems is caused by the contribution of anthropogenic sources such as mining, the use of fertilizers, metal smelting, burning of fossil fuels, manufacturing of batteries, pigments, cement, and plastics, among others. The persistence of each metal in ecosystems varies according to various abiotic and biotic factors that regulate mobility and bioavailability [1,2].

Several methodologies have been proposed to assess toxicity in sediments and the potential risks from heavy metals. Some of these methodologies include sediment quality guidelines and multiple pollution indices such as the enrichment factor, contamination degree, potential ecological risk index, and pollution load index. Many of these methodologies have contributed to understanding the level of contamination and toxicity in coastal environments [3,4]; however, under these evaluation criteria, it is not possible to interpret how these metals enter the food chain. Therefore, in practice, the bioaccumulation factor is evaluated in many marine organisms that may serve as a food source for coastal residents.

Many metals, such as Pb, Cd, Co, Cr, and Cu, are considered dangerous due to their long half-life and high accumulation capacity [5]. Some of these metals, such as Cd, Cr, and Cu, are associated with various health risks in humans, including cancer [6]. Therefore, knowing the concentration of these metals in ecosystems is required to conserve biodiversity and ensure the quality of life for organisms and human health [7]. Although sediments have been the first alternative for measuring the concentration of heavy metals, various benthic organisms are widely used as bioindicators of heavy metal pollution in aquatic systems, such as polychaetes, gastropods, bivalves, and decapod crustaceans [8,9], which helps understand the processes of bioaccumulation and biomagnification in organisms.

The Area for the Protection of Flora and Fauna of the Terminos Lagoon, located in the southeast of Mexico, is one of the ecosystems considered among the most important due to its high biodiversity, its role as a critical habitat for commercially important species, and its importance as a nesting area for sea turtles and migratory birds. It also has great significance because of the abundance of renewable natural resources of commercial interest. However, one of the most important economic activities in the region is oil exploration and exploitation, coupled with human settlements that discharge wastewater into the Laguna de Términos. It is a fact that the threat of contamination is constant due to the anthropogenic activities in the region, which is why a permanent program for monitoring water and sediment pollutants is promoted, with the aim of contributing to proposals that minimize environmental impact and support the development of efficient environmental management.

Within the protected area, there are the Atasta and Pom lagoons, covering an approximate area of 80 km^2^; these lagoons have also been constantly threatened by anthropogenic activities due to the installation of infrastructure for hydrocarbon exploration and transportation, wastewater discharge, deforestation, and exploitation of aquatic species [10]. One of the locally consumed species is the *Rangia cuneata* clam, but currently, the population of this species has significantly decreased at capture sites. This decline has been attributed to two hypotheses: overexploitation of the species and, on the other hand, contamination.

In this regard, there is no evaluation of the toxicity level of sediments and the bioaccumulation factor in *Rangia cuneata*, which would help establish a knowledge base to regulate the consumption of the species and protect its habitat, as sediment acts as a sink for heavy metals, both from natural and anthropogenic sources. Some studies confirm the importance of evaluating the metal concentration in clams and other species, as reported by Romero et al. [11], where the concentration of heavy metals in tissues of *Ariopsis felis* from lagoons adjacent to the Laguna de Términos represents a risk to consumers, particularly children or young individuals aged 8–15 years. This study analyzes the temporal variations in sediments in relation to the concentration of heavy metals, toxicity, and the bioaccumulation factor using the *Rangia cuneata* clam as a bioindicator.

## 2. Materials and Methods

### 2.1. Study Area and Sampling

**Figure 1:**
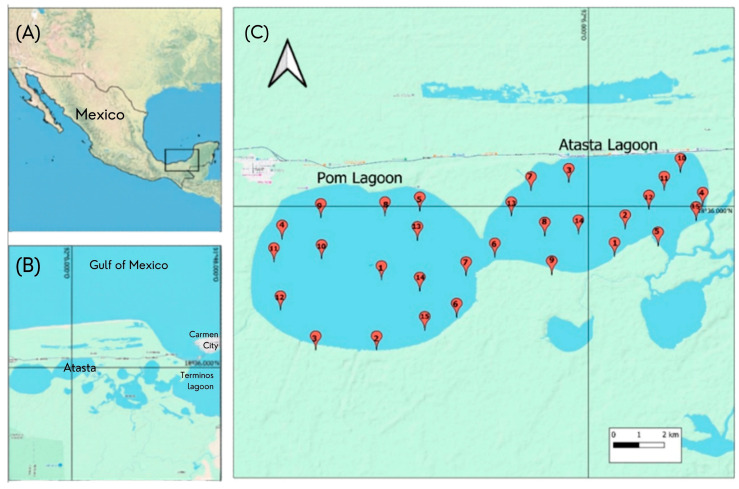
Location and sampling stations for the lagoons Pom and Atasta: the georeferencing process was conducted using ArcGIS 10.8 (Esri, Redlands, CA, USA) with Google Maps. The figure shows (**A**) Location of sampling zone in Mexico, (**B**) Pom-Atasta lagoon system (**C**) Location of sampling stations in Pom-Atasta lagoon.

The study area is located in the western part of the Términos Lagoon between 18°30′ and 18°35′ N and 91°50′ and 92°20′ W within the coastal plain in the state of Campeche (Figure 1). The Atasta and Pom lagoons, with a surface area of 80 km^2^, are connected by a narrow channel, with an average depth for both lagoons of approximately 1.50 m. The tidal flow inputs into the Atasta Lagoon through a meandric marsh that connects with the Términos Lagoon, while the Pom Lagoon receives water from the San Ignacio River. The water mass exchange between the two lagoons contributes to the system’s low salinity of 0.32–3.17 UPS [10].

Sandy-silty sediments, typically deltaic, are the most widely distributed textural group in the Pom–Atasta lagoons. For coastal lagoon systems such as Pom and Atasta, sediments are typically subject to intense hydrodynamic forcing, including tidal exchange, wind-driven resuspension, freshwater inputs during the rainy season, and bioturbation. These processes promote frequent reworking and mixing of the upper sediment layer, which can enhance its responsiveness to recent environmental conditions. The submerged grassland vegetation type is very rare, and mangrove forests predominate around both lagoons [12]. Sediment sampling was carried out throughout the year for three climatic periods: (1) dry season, from February to May; (2) rainy season, from June to September; and (3) north winds, from October to February [13].

A total of 30 stations were located in the Pom and Atasta lagoons in different sections representative of the diverse hydrological conditions (Figure 1); this corresponded to 15 stations for Pom Lagoon and 15 for Atasta Lagoon. Sediment samples were collected from the surface zone (0–10 cm deep) during the dry, rainy, and northerly months using a Van Veen dredge and kept refrigerated (4 °C) until the beginning of the analyses within 24 h. The sediments were oven-dried (Fisher Scientific) at 65 °C, homogenized, and passed through a sieve with a mesh size of <0.02 mm (20 µm sieve) for heavy metal analysis. This method is widely used in surface sediment pollution studies due to its ability to collect representative samples from the active or recently deposited layer. From a geochemical and environmental perspective, the first few centimeters of sediment (generally 0–10 cm) correspond to the most dynamic zone, where key processes such as recent deposition, bioturbation, and exchange with the water column occur. These processes tend to partially homogenize the surface layers of the sediment, reducing vertical variability to centimeter scales. Therefore, concentration differences within this range are expected to have a limited effect on the overall trends observed.

The organism samples were collected near the shoreline of the mangrove area, the habitat of the clam *Rangia cuneata*. Although the species is scarce in the study area, a total of 50 specimens were collected from five sampling stations (approximately 10 organisms per station). Two of these stations were located at the northern end of the lagoons, and three were in the southwestern area.

### 2.2. Sediment Analysis

Sediment characteristics were determined using the Bouyoucos hydrometer technique described in the Mexican standard NOM-021-RECNAT-2000 [14]. This method allows the quantitative determination of particle size distribution of the fine fractions of soils. The distribution of particles larger than 75 µm (retained on the No. 200 sieve) was determined by sieving, while particles smaller than 75 µm were determined by sedimentation using a hydrometer. Therefore, soil texture can be represented as the relative proportion of particle dimensional groups and provides a general overview of sediment physical properties.

Sediment pH was measured in a 1:2.5 (*w*/*v*) ratio of dry sample to deionized water using a multiparameter probe (Hanna HI9829). Organic matter (OM) and total organic carbon (TOC)

$$\%TOC=\left(\frac{B-T}{g}\right)\left(N\right)\left(0.39\right)mcf$$

were determined according to the Walkley and Black method and NOM-021-RECNAT-2000 [14,15]. Briefly, 0.5 g of sediment sieved through a 0.5 mm mesh was treated with 10 mL of 1 N potassium dichromate and 20 mL of concentrated sulfuric acid. After homogenization, 200 mL of distilled water and 5 mL of concentrated phosphoric acid were added, and the solution was titrated with 1 M ferrous sulfate. OM and TOC were calculated using the following equations:

%OM=%TOC×1.724

where *B* is the volume of ferrous sulfate used to titrate the control (mL); T is the volume used to titrate the sample (mL); N is the normality of ferrous sulfate; g is the sample mass (g); mcf is the moisture correction factor.

### 2.3. Metal Analysis

Heavy metal analysis in sediments was performed following EPA method 3051A for microwave-assisted acid digestion and EPA method 200.2 according to protocol SW-846 (US-EPA). For each sample, 0.25 g of dry sediment sieved through a 250 µm mesh was digested using 9 mL of HNO_3_, 3 mL of HCl, and 1 mL of H_2_O_2_ in a microwave digestion system (Mars 6 CEM). Each digestion batch included a blank (BP), a fortified sample (MF), and a certified reference material (CRM). Digestion was conducted for 30 min at 180 °C, and samples were subsequently filtered.

The CRM consisted of BCR^®^-667 estuarine sediment (Institute for Reference Materials and Measurements, European Commission). Fortified samples were prepared by adding 0.25 µL of a multielement standard solution to 0.25 g of sediment prior to digestion. Calibration curves were prepared using standard solutions of Ni, V, Pb, As, Hg, Cd, and Cr. For clam samples, approximately 0.25 g of freeze-dried and homogenized *Rangia cuneata* tissue was digested with 2 mL H_2_O_2_ and 5 mL HNO_3_ and then diluted to 20 mL with Milli-Q water. Samples were filtered through a 0.22 µm syringe filter prior to analysis. Metal concentrations were determined by microwave plasma atomic emission spectrometry (MP-AES 4200; Agilent Technologies Inc 4200, Santa Clara, CA, USA).

#### Estimation of Limit of Detection (LOD) and Limit of Quantification (LOQ)

The estimation of LOD and LOQ was performed following internationally accepted analytical procedures [16]. Calibration curves were constructed using certified reference materials (NIST-SRM-1566b, Merck Brand, National Institute of Standards and Technology, Gaithersburg, MD, USA), with a recovery percentage between 95 and 97%, and blank samples were analyzed to determine background noise. The LOD was defined as LOD = 3 (α)/m and e LOQ = 10 (α)/m. These values ensured the reliability and reproducibility of the heavy metal measurements, where α = 0.00467 represents the standard deviation of the blank readings obtained from the replicates, and m denotes the slope of the calibration curve for each metal. The recovery percentage (% recovery) is reported based on the measurements obtained from the reference standards (Table 1).

### 2.4. Risk Assessment

The ecotoxicology of heavy metals in coastal system sediment was determined through analyses of the Adverse Effects Index (AEI), the Toxic Risk Index (TRI), and the potential ecological risk index (ERI). The AEI was determined by comparing the metal content in samples with sediment quality guidelines, thereby analyzing the metal levels exceeded in the samples compared to the guideline values. The AEI value was calculated using the following equation [3]:

$$AEI=\sum_{i=1}^{n}\frac{{Cs}^{i}}{C_{PEL}^{i}}$$

where ${C_{s}}^{i}$ is the metal concentration in the sediment sample (µg g^−1^), and $C_{PEL}^{i}$ was selected from the sediment quality guideline (µg g^−1^) as the threshold effect level (TEL) and the probable effect level (PEL). In the present study, the PEL was used to evaluate the sediment–biota effect level [17]. The criteria for AEI are as follows: values of AEI < 1.0 mean that the metal concentration in the sample is not large enough to produce adverse effects on the biota; conversely, an AEI ≥ 1.0 indicates that the metal concentration could produce effects on sedimentary fauna [3].

To better approximate the toxic risk (TRI), both the PEL ($C_{PEL}^{i}$) and TEL ($C_{TEL}^{i}$) effects of metal *i* were considered [18,19], and they are described by the following equation. The toxic risk was classified into five categories based on the TRI values (Table 2).

TRI=∑i=1nTRIi=CsiCTELi2+CsiCPELi22

The potential ecological risk index (ERI) was determined by summing the risk factors for all heavy metals in sediments, and it represents the sensitivity of the biological community to toxic substances [20]. Because the ERI can assess the effects of multiple contaminants on an ecosystem simultaneously, this method is very practical [2,21]. The risk potential can be determined using the following equations:

$$ERI=\sum E_{r}^{i}$$

$$E_{r}^{i}=T_{r}^{i}\;\times \;{Cf}^{i}$$

$$Cf=\frac{Cs}{Cr}$$

$$C_{d}=\sum {Cf}^{i}$$

where Cs and Cr are the metal concentration in the sediment sample (µg g^−1^) and reference value (µg g^−1^): (As = 15, Pb = 70, Cr = 90, Ni = 57, V = 99). $T_{r}^{i}$ is the biological toxic response factor of an individual element (As = 10, Pb = 5, Cr = 2, Ni = 5, V = 2); $E_{r}^{i}\;$ is the ecological risk factor of an individual metal, classified as follows: values less than 40 suggest low ecological risk, values from 40 to 80 denote moderate risk, values from 80 to 160 denote considerable risk, values from 160 to 320 denote high ecological risk, and values >320 denote very high risk. While the ERI is the sum of $E_{r}^{i}$, ERI values less than 150 indicate low ecological risk; in contrast, ERI values in the range of 150–300, 300–600, and >600 are considered moderate, considerable, and very high risk, respectively. C_d_ is the comprehensive pollution index of multicontaminants, which represents the degree of pollution described in Table 2.

### 2.5. Biota–Sediment Accumulation Factor (BSAF)

The bioaccumulation factor (BSAF) used here for the clam *Rangia cuneata* allows us to understand the influence of sediments on aquatic organisms and to quantify the ability of the clam *Rangia cuneata* to digest or eliminate heavy metals; therefore, a high BSAF value indicates a low ability to digest and eliminate contaminants (high bioaccumulation). The BSAF determination was calculated using the following equation:

BSAF=CorganismCsediment

where C_organism_ is the concentration of heavy metals in *Rangia cuneata*, and C_sediment_ is the concentration of metals in sediment.

### 2.6. Statistical Analysis

One-way analysis of variance (ANOVA) with Duncan’s test was applied to determine the significant difference between the total concentration of trace metal and the potential ecological risk of the sediment sample (*p* < 0.05) for the three climatic seasons and between lagoons; a post hoc Tukey test was considered to indicate a significant difference. The determination coefficient (R^2^) was derived from Pearson correlation analysis between the heavy metal concentration in the clam and sediment.

## 3. Results and Discussions

### 3.1. Sediment Texture and Organic Content

Sediment particle size distribution (sand, silt, and clay) was analyzed following standard procedures. Statistically significant differences were observed in sand and silt content across seasons (ANOVA, *p* ≤ 0.01), whereas clay content showed no significant variation (*p* = 0.172). Tukey’s post hoc test revealed that both sand (*p* = 0.014) and silt (*p* = 0.007) contents were significantly different during the northerly season compared to the dry and rainy seasons (Table 3).

The organic matter (OM) and total organic carbon (TOC) contents did not differ significantly across seasons (*p* = 0.172). In both lagoons, sediment composition followed the order sand > silt > clay. OM and TOC content remained relatively consistent, with values ranging from 2.97% to 3.94% and 1.72% to 2.28%, respectively (Table 3).

According to Flemming [22], the high sand and silt content observed in the study area suggests a high ecosystem dynamic with a strong influence of marine and river currents that contribute to the transport and deposition of these materials, which are high-energy hydrodynamic conditions typical of lotic environments. On the other hand, high OM contents were observed mainly in the vicinity of the towns at the following stations: A3 (from 4.41% to 4.69%); A4 (from 5.86% to 11.4%); A7 (from 5.86% to 8.55%); A10 (from 3.74% to 3.90%); A13 (from 7.37% to 9.89%); and P14 (from 4.81% to 7.76%). This suggests a possible contribution of wastewater, as reported by Ruiz-Marin et al. [10], which contributes significantly to the increase in organic carbon in sediment through the input of domestic and industrial wastewater; however, the TOC content obtained from both the Pom and Atasta lagoons did not exceed the 10% limit according to the sediment quality guidelines of the Ontario Ministry of Environment and Energy-1993 [19], suggesting a level of sediment contamination that can be tolerated by the majority of benthic organisms. It is important to mention that, although the 0–10 cm surface layer is widely used to characterize recent sediment conditions, it may integrate material deposited over multiple years, particularly in environments with low sedimentation rates. In coastal lagoon systems such as Pom and Atasta, however, the upper sediment layer is frequently reworked by hydrodynamic processes (tidal action, wind-induced resuspension, and freshwater inputs) and biological activity (bioturbation), which promotes mixing and reduces vertical stratification. Therefore, the sediment samples analyzed in this study are interpreted as representing recently reworked surface material influenced by prevailing seasonal conditions [23].

### 3.2. Heavy Metal Concentrations in Sediment and Bioaccumulation in Mollusk

Trace metal analysis in sediment from the Pom–Atasta lagoon system showed values ranging from Cr (115.0 to 130.4 µg g^−1^) > Ni (60.5 to 75.9 µg g^−1^) > V (32.0 to 35.7 µg g^−1^) > As (12.7 to 15.4 µg g^−1^) > Pb (8.3 to 14.7 µg g^−1^) (Table 4). The concentrations of heavy metals exceeding the reference values for the quality of marine sediments and the ISQG-TEL threshold effect were As, Cr, and Ni, indicating ecological risk and the accumulation of these metals in the sediment due to anthropogenic activities (Table 4).

**Figure 2:**
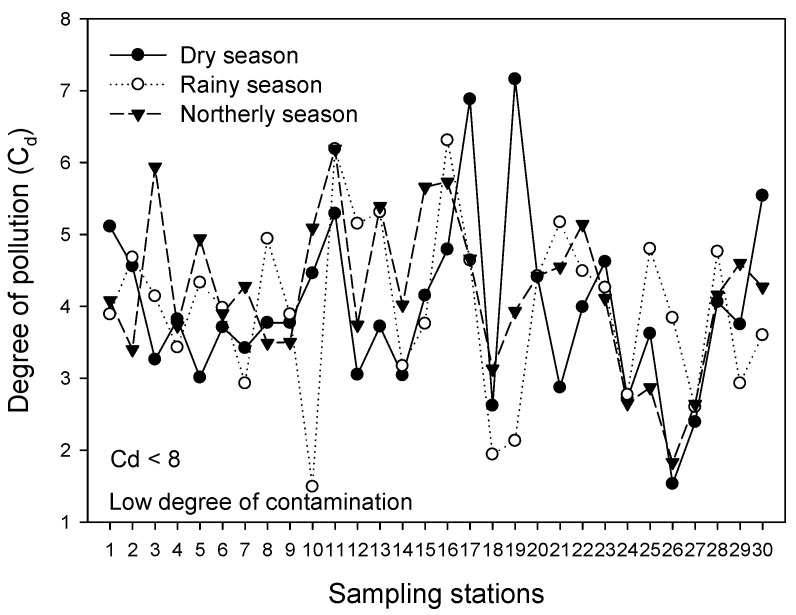
Degree of contamination (Cd) by heavy metals in sediment during the climatic seasons for the Pom–Atasta fluvial lagoon system.

Similarly, based on the reference values reported by Wedepohl [24], the content of As, Cr, and Ni in sediment was high, indicating metal enrichment from anthropogenic sources, while the low concentrations of Pb and V relative to the values in the upper crust suggest an origin from the Earth’s crust (Table 4). However, it is likely that Pb and V have a historical origin due to the manufacture of batteries, paints, biocides, fertilizers, plastics, refineries, and fuels, activities that had little environmental regulation in the past years. Overall, both lagoons were evaluated to have a low degree of contamination (Cd < 8) by heavy metals in sediment during the climatic seasons (Figure 2).

The oil industry’s role in the enrichment of Ni and V in ecosystems is recognized through contributions from hydrocarbon spills, where trace elements such as Fe, Ni, V, and Zn are inorganic components present in crude oil, with Ni and V being the most abundant [25]. The Terminos Lagoon protected natural area and its fluvial-lagoon systems are located in one of the most productive oil and gas extraction and exploration areas in Mexico. Although the impact of these activities on adjacent ecosystems has been assessed, few restoration measures have been implemented. According to Magallanes-Ordoñez et al. [26], sediments enriched with Ni and V can occur naturally when these metals are related to terrigenous elements (rock weathering). In contrast, in ecosystems near hydrocarbon and gas exploration zones, the relationship with terrigenous elements is null.

Thus, to define the possible source of Ni and V enrichment in the study area, the Ni/V ratio was used as an empirical tool for correlation with the type of hydrocarbon in sediments. The criteria suggest that a low ratio (Ni/V < 0.5) indicates a hydrocarbon origin from marine organic matter, while high values (1 < Ni/V < 10) are derived from crude oil for which its organic matter is of terrestrial or lacustrine origin [27]. In the present study, a significant correlation between Ni and V was observed during the dry, rainy, and northerly seasons (r = 0.61, r = 0.63, and r = 0.74, respectively), indicating that these elements have a similar source, unlike the rest of the heavy metals analyzed. Thus, the estimated Ni/V ratios for the three climatic seasons were > 1 (1.8, 2.13, and 2.12, respectively), similar to that reported for the Términos Lagoon and the coastal zone of Campeche (Mexico), as well as other regions of the world (Table 5). The results suggest that the probable origin is probably attributed to urban and industrial wastewater discharges [28]. The results show evidence that oil activities near the two lagoons do not fully contribute to the enrichment of the sediment by Ni and V; therefore, natural biogeochemical processes characteristic of lacustrine systems likely also contribute.

The Ni/V ratio alone is insufficient to definitively exclude hydrocarbons as a dominant source of contamination. While this ratio often suggests a stronger influence from urban and industrial wastewater, the presence of regional petroleum-related activities indicates that hydrocarbons remain a relevant environmental pressure and a potential source of heavy metal pollution. Therefore, the Ni/V ratio should be interpreted with caution, as it is neither exclusive nor conclusive in source attribution. This interpretation is supported by studies such as Yakubov et al. [29], which examined V and Ni concentrations in heavy oil asphaltenes and highlighted the variability of Ni/V ratios across petroleum sources. Their findings emphasize that although Ni/V ratios can help distinguish between anthropogenic inputs, they do not provide absolute source identification.

This is also supported by the low concentration ranges for Ni (from 60.5 to 75.9 µg g^−1^) and V (from 32.0 to 35.7 µg g^−1^) in both the Pom and Atasta lagoons (Table 4), which do not greatly exceed the maximum limit concentrations of Ni (60 µg g^−1^) and V (211 µg g^−1^) used to classify an ecosystem contaminated by accidental spills as a result of from oil production in coastal areas [30].

**Table 5:** Ni/V concentration and ratio reported on the coasts of Campeche and Terminos Lagoon, Mexico.

Location	Ni (µg g^−1^)	V (µg g^−1^)	Rate Ni/V	Reference
Jinjiang River, China	0.51–2.85 Media: 1.70	0.53–1.91 Media: 0.97	1.75	[28]
Terminos Lagoon, México	13.5–164 Media: 88.75	9–61 Media: 35	2.53	[26]
Terminos Lagoon, México	16.29–56.45 Media: 20.08	nd	-	[31]
Campeche Coast, Mexico	0.56–76.9 Media: 38.73	15.6–117.5 Media: 66.55	0.58	[32]
Campeche and Tabasco coast, Mexico	1.54–211 Media: 106.27	17.7–59.6 Media: 38.65	2.74	[30]
Atasta Lagoon, Mexico	59.56–78.39 Media: 68.97	32.15–40.67 Media: 36.41	1.89	This study
Pom Lagoon, Mexico	61.37–73.43 Media: 67.4	30.70–33.1 Media: 31.9	2.11	This study

n.d.: Not detected.

In the present study, the highest concentration of metals in sediment occurs during the rainy and northerly seasons (Table 4), attributed to the increase in rainfall runoff, fluvial contributions, and sediment resuspension [33]. The low correlation of As, Cr, Pb, and Cd, unlike Ni and V (Table 6), suggests that these metals could be transported to the Pom and Atasta lagoons, having origins both from natural contributions and anthropogenic activities. From a comparative analysis of the concentration of metals in other ecosystems, it is possible to observe that the levels of As, Cr, Pb, and V obtained in the present work were generally lower than those reported in other studies; however, for this study, Ni and Cr were found in higher concentrations than reported by Zang and Liu [34], with Cr levels of 48.08–107.0 µg g^−1^; likewise, they were found in higher concentrations than those reported by Liu et al. [27], with Cr and Ni levels of 39.3–92.1 µg g^−1^ and 17.2–41.4 µg g^−1^, respectively, and those reported by Norville [35], with Cr and Ni levels of 19.2 and 4.8–15.9 µg g^−1^, respectively. Similarly, Saher and Siddiqui [2] report Cr and Ni levels of 144.80 and 40.02 µg g^−1^, respectively, suggesting an enrichment of Cr and Ni for both lagoons (Pom and Atasta) from various anthropogenic sources, including urban and industrial wastewater discharges.

On the other hand, the concentration ranges of heavy metals in the tissues of the clam *Rangia cuneata* followed the order Ni (21.36 µg g^−1^) > Cr (9.48 µg g^−1^) > Hg (0.51 µg g^−1^) > Cd (0.43 µg g^−1^) > As (<0.3 µg g^−1^) > Pb (<1 µg g^−1^) (Table 4). Tissue metal levels showed a weak correlation with Ni, Cr, As, and Pb concentrations in sediments. Notably, Cd and Hg detected in *Rangia cuneata* tissues were not observed in sediments. The detection of Cd and Hg in tissues, in the absence of measurable concentrations in surrounding sediments, suggests the contribution of alternative exposure pathways, potentially including the water column. As filter feeders, bivalves are continuously exposed to suspended particles that may be enriched with heavy metals, even in environments where sediment contamination is minimal or undetectable. This interpretation is consistent with Zhang et al. [36], who reported that metal concentrations in bivalve tissues were more strongly associated with waterborne contamination than with sediment levels in Haizhou Bay, China. Furthermore, the weak correlation between sediment and tissue metal levels in *Rangia cuneata* supports the idea that bioaccumulation is not solely driven by sediment-associated exposure but may involve multiple pathways, including filtration of the water column, dietary uptake, and trophic transfer. Similarly, Hao et al. [33] highlighted that filter-feeding organisms have a high capacity to accumulate contaminants bound to organic matter or dissolved in water compared to other benthic organisms or fish.

In the present study, the bioaccumulation order for *Rangia cuneata* was Ni > Pb > Cr > As. It is worth mentioning that, although Cr was the metal with the highest concentration in sediment, it showed a bioaccumulation factor of SBAF < 1, while greater bioaccumulation was observed for Ni (SBAF > 1), which was the second metal in order of lowest concentration in the sediment (Table 4). Several bivalve species, including *Rangia cuneata*, exhibit selective absorption of heavy metals, with a notable tendency to accumulate Ni in higher concentrations than Cr. This differential absorption is influenced by both environmental availability and physiological mechanisms [37]. Ni generally exhibits greater bioavailability in aquatic environments than trivalent chromium (Cr (III)), which is less soluble and therefore less readily absorbed by filter-feeding organisms. The higher solubility of Ni allows it to remain in ionic form, facilitating its interaction with biological membranes and subsequent uptake into soft tissues. Additionally, the resuspension of sediments may further enhance metal bioavailability by releasing particle-bound metals into the water column, increasing their accessibility to filter-feeding organisms such as *Rangia cuneata.* Furthermore, clams possess specific physiological adaptations that enhance the absorption of certain metals. Ni has a strong affinity for metallothioneins and other cellular proteins, allowing it to bind efficiently to tissues. This binding capacity contributes to its accumulation [38]. It is estimated that marine organisms in environments with high concentrations of heavy metals generally have high SBAF values; however, this was not possible to observe in the present study, suggesting that the bioaccumulation of heavy metals by *Rangia cuneata* is mainly related to the environmental background values, which contribute more to the distribution of heavy metals in the ecosystem and therefore to greater exposure. This indicates that a heavy metal may be present in high concentrations but only in a limited area, consequently showing low bioaccumulation ability, contrary to heavy metals that have greater dispersion or distribution in the sediments [33].

### 3.3. Heavy Metal Toxicity Assessment

**Figure 3:**
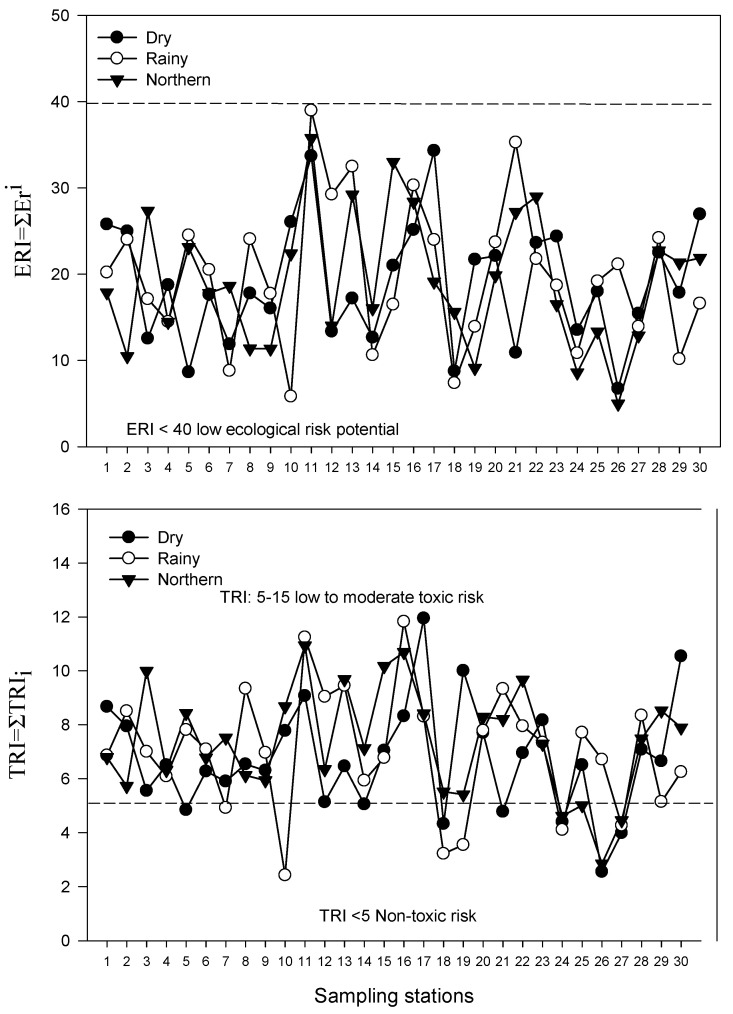
Ecological risk level (ERI) and toxic potential (TRI) in sediments of the Pom–Atasta fluvial lagoon system.

The ecotoxicological profile analysis for the study area was determined using the adverse effect index (AEI) and the potential ecological risk index (TRI). Metals such as As, Cr, Pb, V, and Cd showed an AEI < 1, indicating that the concentrations of these metals in the sediment of both lagoons during the climatic seasons are not high enough to produce adverse effects on the biota; meanwhile, Ni showed values for both lagoons that exceeded the AEI > 1 criterion, suggesting that Ni could produce effects on the sedimentary fauna. This is related to the metal that showed the highest biaccumulation factor (BSAF = 1.51) (Table 4). This could indicate that the Ni concentration is likely to affect the sedimentary environment, creating an unsuitable environment for the clam *Rangia cuneata* to increase its population in both lagoons. Based on the TRI indicator, both lagoons show heavy metal concentrations with a low to moderate toxic risk potential and a low ecological risk potential (ERI) (Figure 3). It was evident that Ni contributes to increasing ecological risk mainly for sedimentary fauna, and therefore, a permanent monitoring program should be considered in the study area to prevent possible impacts on other organisms.

## 4. Conclusions

The results of this study reveal significant seasonal variability in the concentration of heavy metals in the sediments of the Pom–Atasta lagoon system, with higher levels observed during the rainy season and periods of northern winds. This variation is likely driven by increased riverine inputs, surface runoff, and sediment resuspension, which facilitate the transport and deposition of metals such as chromium (Cr), nickel (Ni), and vanadium (V). The lack of significant correlation among certain metals (arsenic [As], Cr, lead [Pb], and cadmium [Cd]) suggests multiple sources of origin, both natural and anthropogenic. Although overall concentrations of As, Cr, Pb, and V were lower than those reported in other estuarine systems, a notable enrichment of Cr and Ni was detected in both lagoon bodies, indicating the influence of urban and industrial discharges.

Nickel, in particular, exhibited the highest bioaccumulation factor (BSAF > 1) in *Rangia cuneata*, despite not being the most abundant metal in the sediment. This reinforces the notion that bioaccumulation is more closely linked to metal bioavailability and environmental distribution than to total sediment concentration. Ecotoxicological assessment using the AEI (Aquatic Ecotoxicity Index) and TRI (Toxic Risk Index) indicated that most metals pose a low to moderate risk to aquatic biota, with the exception of nickel, which exceeded ecological risk thresholds and represents a potential threat to benthic fauna.

TRI and ERI (Ecological Risk Index) values indicating low to moderate risk may have implications for local fisheries and food safety, particularly if *Rangia cuneata* is consumed by nearby populations. While these values do not signal an immediate health alert, they highlight the need for continuous monitoring to detect changes in the distribution and toxicity of heavy metals, especially those like Ni, which exhibit high mobility and bioaccumulation potential in filter-feeding organisms.

Overall, this study provides robust evidence of the seasonal dynamics, sources, and ecological risks of heavy metals in a tropical river–lagoon system, contributing to a deeper understanding of their environmental impact and serving as a foundation for the development of environmental management strategies and pollution control measures.

## Figures and Tables

**Table 1:** Estimation of LOD and LOQ parameters and recovery percentages in µg g^−1^.

Metals	LOD	LOQ	Recovery (%)
Cr	0.12	0.40	96.16
Ni	0.024	0.08	95.06
V	0.018	0.06	95.86
As	0.021	0.07	97.58
Pb	0.12	0.40	94.60
Cd	0.018	0.06	96.56

**Table 2:** TRI and C_d_ criteria for the level of heavy metal contamination in sediment.

TRI	Toxic Risk	C_d_	Degree of Contamination
<5	No	C_d_ < 8	Low
5–10	Low	8 ≤ C_d_ < 16	Moderate
10–15	Moderate	16 ≤ C_d_ < 32	Considerable
15–20	Considerable	C_d_ ≥ 32	Very high
>20	Very high		

**Table 3:** Sediment texture, organic matter (OM), and organic carbon (TOC) in the Pom–Atasta lagoon system across climatic seasons (mean ± SD).

Climate Season	Sand (%)	Silt (%)	Clay (%)	OM (%)	TOC (%)
Dry	76.47 ± 8.89 ^a^	19.73 ± 8.55 ^a^	3.79 ± 1.56 ^a^	3.94 ± 2.13 ^a^	2.28 ± 1.24 ^a^
Rain	73.96 ± 9.81 ^a^	22.73 ± 10.02 ^a^	3.31 ± 1.65 ^a^	2.97 ± 2.21 ^a^	1.72 ± 1.28 ^a^
Northerly	67.27 ± 16.74 ^b^	29.59 ± 16.54 ^b^	3.14 ± 1.14 ^a^	3.03 ± 2.33 ^a^	1.76 ± 1.35 ^a^

^a,b^ = Different letters indicate significant differences (*p* < 0.05).

**Table 4:** Average concentration of heavy metals in sediment, ISQG-TEL, and upper continental crust (UC). Metals in *Rangia cuneata* (±SD) and sediment–biota accumulation factor (SBAF).

Metal (µg g^−1^)	Clam		Sediment Climatic Seasons				UC
	*Rangia* *cuneata*	SBAF	Dry	Rains	Northern	ISQG-TEL	Wedepohl [24]
As	<0.3	0.14	15.4 ± 8.9 ^a^	16.0 ± 10.6 ^a^	12.7 ± 9.8 ^a^	5.9	2.0
Cr	9.48 ± 3.36	0.37	128.3 ± 72.5 ^b^	115.0 ± 29.5 ^b^	130.4 ± 37 ^b^	52.3	35
Ni	21.36 ± 6.55	1.51	60.5 ± 22.4 ^c^	68.2 ± 27.4 ^c^	75.9 ± 22.8 ^d^	15.9	18.6
Pb	<1	0.65	8.3 ± 2.9 ^e^	9.3 ± 5.3 ^e^	14.7 ± 5.2 ^f^	30.2	17
V	nd	-	33.6 ± 7.6 ^g^	32.0 ± 8.9 ^g^	35.7 ± 8.8 ^g^	-	53
Cd	0.44	-	nd	nd	nd	0.68	0.102
Hg	0.51	-	nd	nd	nd	-	-

Different letters (letter superscript from a to g) show significant differences (Tukey *p* ≤ 0.05); nd: not detected.

**Table 6:** The Pearson’s correlation coefficients of trace metals in sediment.

		1As	1Cr	1Ni	1Pb	1V	2As	2Cr	2Ni	2Pb	2V	3As	3Cr	3Ni	3Pb	3V
Dry	1As	1														
	1Cr	0.03	1													
	1Ni	0.26	0.06	1												
	1Pb	0	−0.09	0.32	1											
	1V	0.28	0.39	0.82	0.27	1										
Rain	2As	0.23	0.04	−0.07	0.03	0.03	1									
	2Cr	0.12	−0.33	0.28	−0.01	0.21	0.04	1								
	2Ni	0.24	−0.22	0.38	0.31	0.35	0.23	0.47	1							
	2Pb	0.09	−0.32	0.14	−0.02	0.01	0.08	0.34	0.11	1						
	2V	0.21	−0.3	0.45	0.14	0.37	0.16	0.62	0.88	0.42	1					
Northerly	3As	0.29	−0.19	0.14	0.02	0.13	0.4	0.09	0.2	0.1	0.13	1				
	3Cr	0.05	0.8	−0.04	−0.03	0.3	0.04	−0.25	−0.18	−0.34	−0.33	−0.1	1			
	3Ni	0.34	−0.18	0.6	0.38	0.61	0.01	0.29	0.67	0.2	0.63	0.4	−0.06	1		
	3Pb	0.06	−0.15	0.21	0.48	0.37	−0.11	0.25	0.4	0.23	0.36	0.13	0.11	0.58	1	
	3V	0.19	0.07	0.44	0.37	0.62	−0.06	0.2	0.54	−0.02	0.43	0.2	0.37	0.74	0.8	1

Blue and red colors show low and high correlation coefficients (R) values, respectively.

## Data Availability

The original contributions presented in this study are included in the article. Further inquiries can be directed to the corresponding author.

## Abbreviations

The following abbreviations are used in this manuscript:

AEI	Adverse effect index;
TRI	Toxic risk index;
ISQG-TEL	Reference values for the quality of marine sediments and threshold effect;
BSAF	Biota–sediment accumulation factor.
